# In Vitro Inhibiting Effects of Three Fungal Species on Eggs of Donkey Gastrointestinal Strongyles

**DOI:** 10.3390/vetsci7020053

**Published:** 2020-04-25

**Authors:** Michela Maestrini, Simona Nardoni, Francesca Mancianti, Simone Mancini, Stefania Perrucci

**Affiliations:** Department of Veterinary Sciences, University of Pisa, Viale delle Piagge 2, 56124 Pisa, Italy; michela.maestrini@phd.unipi.it (M.M.); simona.nardoni@unipi.it (S.N.); francesca.mancianti@unipi.it (F.M.); simone.mancini@unipi.it (S.M.)

**Keywords:** gastrointestinal nematodes, donkey, chitin degrading fungi, ovicidal activity, biological control

## Abstract

Recently, donkeys have gained popularity mainly due to the use of donkey milk by the cosmetic industry and for human consumption. Gastrointestinal strongyles (GIS) are considered a potential cause of disease and reduced production in infected donkeys. European laws limit the use of anthelmintic drugs for the control of GIS in dairy donkey farms, thus the need to develop alternative control methods. This study aimed to test the in vitro inhibiting effects of three chitin degrading fungi (*Scopulariopsis brevicaulis*, *Metarhizium anisopliae*, and *Beauveria bassiana*) on the hatch and viability of donkey GIS eggs by using the egg hatch test, and to compare their activity to that of *Pochonia chlamydosporia*. About 150 eggs were added to 0.5 mL of sterile saline solution containing about 1.4 × 10^8^ spores of each fungal species or with 0.5 mL of sterile saline solution only (untreated controls). After incubation, the percentage of egg hatch reduction was calculated, and data were statistically analyzed. All fungi were able to significantly reduce (*p* < 0.05) the hatch of GIS eggs compared to the untreated controls. Further studies that aim to investigate the efficiency of these fungi in reducing donkey GIS eggs in contaminated environments are encouraged.

## 1. Introduction

In recent years, the interest in dairy donkey farming has increased considerably in relation to the use of donkeys for social activities and to the popularity gained by donkey milk in some European countries [1]. In Italy, donkey milk is used for human consumption, and about 35 dairy farms and 700 donkeys of different breeds can be found in this country [1]. Asinine milk has many interesting features, including the similarity to breast milk, which makes this milk widely used in neonatal nutrition because it is considered a valid substitute for children allergic to cow milk [2]. Due to its high content in essential fatty acids with antioxidant properties and several vitamins (A, B, C, and E) which play an epithelial-protecting role, donkey milk is also used in cosmetics [3]. In order to guarantee the quantity, quality, and safety of donkey milk, it is necessary to adequately control donkey pathogens. Among pathogens, helminth infections are frequently observed in donkeys and may negatively affect the health and the productive performances of infected animals [4]. Gastrointestinal strongyles (GIS), especially cyathostomins, are considered some of the most common and important donkey helminths [4,5]. In horses, the administration of anthelmintic drugs, mainly benzimidazoles, macrocyclic lactones, and tetrahydropyrimidines, may substantially reduce the prevalence of GIS infections [6,7]. Nevertheless, the control of GIS in dairy donkey farms may be problematic. Firstly, anthelmintics commercially available for horses are not always licensed and safe to be used in donkeys [5], despite some recent studies showing the efficacy and safety of two oral pyrantel pamoate formulations and a mebendazole oral paste against donkey gastrointestinal strongyles [8,9]. A minimal disposition rate into donkey milk of the mebendazole paste has been also reported [9]. However, in dairy donkeys the use of drugs needs to be carefully managed according to the limitations of EC regulations (853/2004/EC), especially in organic farms (470/2009/EC and 37/2010/EC). For all these reasons, alternative methods for the control of donkey GIS are urgently needed. Among the various alternative methods for the control of equine nematodes, fecal removal from pastures [10], pasture rotation [11], administration of food supplements [12], phytotherapy [13], and biological control [11] are included. Biological control methods for nematode parasites include the use of nematophagous fungi [14]. Based on predatory modes, these fungi can be divided into three groups: endoparasites, predators, and ovicidal or opportunists [15]. All these fungi have a common characteristic, namely, the ability to degrade chitin. This biopolymer is a fundamental component of the cell wall of fungi and of the cuticle of arthropods and helminths [16]. The ovicidal activity on helminth eggs in most cases consists of the penetration of the hyphae inside the small pores present on the surface of the eggs, which causes a change in the permeability of the shell, resulting in an increase in the egg volume. The hyphae increase in size and expand through the adjacent layer of chitin and lipids with consequent separation of the yolk sac, vacuolation of the chitin layer, and dispersion of lipids [15]. 

Aiming to find a biological method for the control of donkey GIS, the ability of the fungi *Scopulariopsis brevicaulis*, *Beauveria bassiana,* and *Metarhizium anisopliae* to inhibit the hatch of donkey GIS eggs is evaluated in vitro. Fungi are selected based on their chitin degrading and antiparasitic properties reported in literature [17,18,19]. *P. chlamydosporia*, whose ovicidal activity on nematode eggs is already known [20,21], is chosen as a model to be compared against the inhibiting activity of the other selected fungi.

## 2. Materials and Methods

### 2.1. Fungi

Reference strains (Westerdijk Fungal Biodiversity Institute, Utrecht, The Netherlands) of *P. chlamydosporia*, *S. brevicaulis*, *M. anisopliae,* and *B. bassiana* were used in this study. Fungi were grown on Malt Agar Medium, then incubated at 25 °C in dark conditions, until a mycelial growth was noticeable. Fungal inocula, each consisting of about 1.4 × 10^8^ spores of each fungal species in 0.5 mL of sterile saline solution, were prepared and used in the assays performed in this study.

### 2.2. Sampling

Individual fecal samples were collected from the rectal ampulla of Amiatina donkeys naturally infected by GIS and living in a farm in Central Italy. The farm hosts about 160 donkeys that are bred for onotherapy, trekking, and the production of milk for pediatric and geriatric human use according to EC regulation 853/2004/EC.

### 2.3. Parasitological Analysis and Recovery, Suspension, and Purification of Gastrointestinal Strongyle (GIS) Eggs

Collected fecal samples (n = 30) were analyzed using a McMaster technique with a sensitivity of 50 eggs per gram of feces (EPG) [22]. Only positive samples with more than 1000 EPG of GIS eggs were used for the in vitro experiments. For the concentration and purification of GIS eggs, a previously reported technique was used on pooled fecal samples (each consisting of 3–4 individuals) [23]. Briefly, 30 g of fecal material was homogenated with distilled water, and the resulted suspension was sieved, placed in 50 mL tubes, and centrifuged for 5 min at 1000× *g*. The sediment was collected, and the pellet suspended in a NaCl saturated solution (specific gravity 1.2) and centrifuged for 5 min at 1000× *g*. The supernatant was then collected, diluted in distilled water in 15 mL tubes, and then centrifuged for a further 2 min at 1000× *g*. The sediment obtained with this last centrifugation, containing the eggs, was collected and diluted in sterile saline solution, and the number of GIS eggs/mL of suspension was counted. The eggs were then used for the evaluation of the in vitro ovicidal activity of tested fungi.

### 2.4. Egg Hatch Test (EHT)

The EHT according to the method reported by Coles et al. [23] was the test used in this study to assess the in vitro ovicidal efficacy of selected fungi on GIS eggs. This test consists of evaluating the inhibition of the hatching and/or the development of GIS eggs by a tested compound, compared to untreated control eggs [23]. All experiments were performed in triplicate in three independent assays. An amount of 0.5 mL of sterile saline solution containing about 150 eggs and about 1.4 × 10^8^ spores of each fungal species in 0.5 mL of sterile saline solution was placed in each well of 24-well plates (TC Plate 24 Well, Standard, F, SARSTEDT S.r.l., Verona, Italy). In controls, each well contained 0.5 mL of the egg suspension and 0.5 mL of sterile saline solution. After a 48-h incubation in the dark, at 25 °C and 90% relative humidity, plates were observed under a stereoscope microscope (Leica M165 C, Leica Microsystems S.r.l., Milan, Italy) and the number of eggs and of first-stage larvae (L1) in each well was counted. The percentage of egg hatch reduction was calculated using the following formula [24]:

number of eggs/(number of L1 + number of eggs) × 100

Micromorphological alterations of GIS eggs were recorded. More specifically, the growth of fungal hyphae on the eggshell and the eventual penetration of fungal hyphae into the shell in treated eggs compared to control eggs represented the main parameters assessed under an optical microscope.

### 2.5. Identification of GIS Genera

The identification of GIS genera was carried out by setting up of fecal cultures from pooled samples. More specifically, about 20 g of feces was placed on a gauze stretched inside a plastic cup containing about 20 mL of water and covered with the bottom of a perforated plastic cup to allow proper oxygenation. Fecal cultures were incubated at 26–28 °C for 7 days. During the incubation period, fecal cultures were sprayed with water daily to ensure adequate humidity conditions [25]. After this period, L3 larvae were obtained using the Baermann technique, and they were morphologically identified at the genus/species level according to reference keys [25,26].

### 2.6. Statistical Analysis

The inhibiting effects of the fungi *S. brevicaulis*, *B. bassiana,* and *M. anisopliae* on the hatch of GIS eggs, expressed as percentage of egg hatch reduction, were statistically analyzed by one-way ANOVA test against the control samples (untreated, negative control) and *P. chlamydosporia* (positive control) egg hatch percentages. The significance was set at 5% (*p* < 0.05), and the eventual significance was tested with post-hoc Tukey test. Statistical analysis was carried out using the Statistical Analysis System (SAS) program. 

## 3. Results

**Figure 1:**
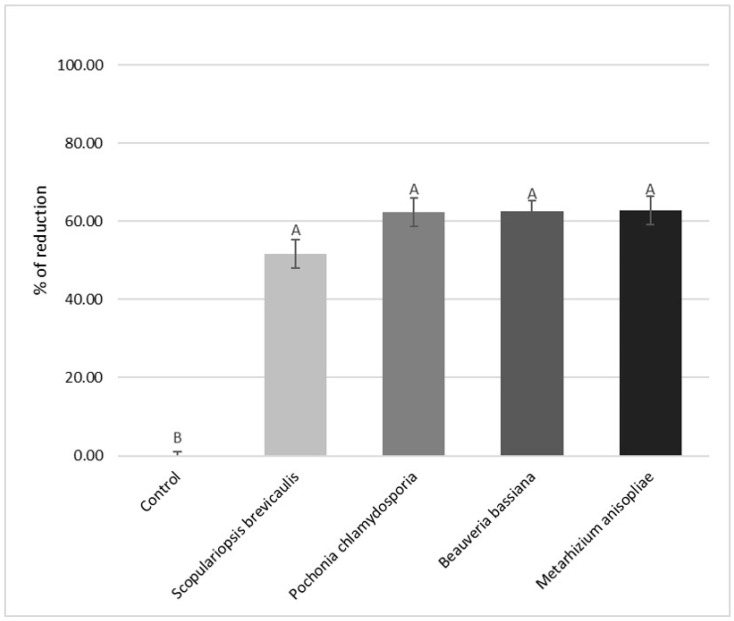
Egg hatch reduction percentages on gastrointestinal strongyles GIS eggs observed for *Scopulariopsis brevicaulis*, *Beauveria bassiana,* and *Metarhizium anisopliae,* compared to that of *Pochonia chlamydosporia* and the untreated control. Vertical bars indicate the standard deviation. ^A, B^ Different letters indicate significant differences at *p* < 0.05.

*M. anisopliae* and *B. bassiana* were able to significantly reduce (*p* < 0.05) the hatching of eggs (62.8% ± 3.88 and 62.2% ± 4.76, respectively) when compared to the untreated controls (Figure 1), while no differences were evidenced from the comparison of activity of these fungi to that of *P. chlamydosporia* (62.6% ± 3.89). *S. brevicaulis* (Figure 1) showed a lower efficiency than the other tested fungi, since it caused an egg hatch reduction of about 52% ± 3.88. However, this difference was not statistically significant when compared to that of other tested fungi, while significant negative effects of *S. brevicaulis* on GIS egg hatch emerged from the comparison with the untreated controls (*p* < 0.05).

**Figure 2:**
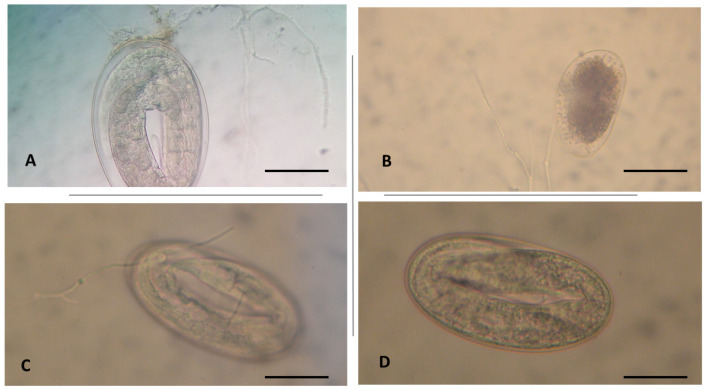
Colonization and penetration of fungal hyphae through the eggshell of donkey gastrointestinal strongyle eggs by (**A**) *Scopulariopsis brevicaulis* (400×; scale bar 32 µm), (**B**) *Metarhizium anisopliae* (250×; scale bar 60 µm), and (**C**) *Pochonia chalmydosporia* (400×; scale bar 32 µm), compared to untreated eggs (**D**) (100×; scale bar 30 µm).

At microscopical examination, eggshell colonization by fungal hyphae was also observed (Figure 2).

Identification of GIS genera by pooled coprocultures revealed a high prevalence (>90%) of cyathostomins. More specifically, species belonging to the genera *Cylicocyclus* and *Cylicostephanus* were identified. The remaining 10% of the identified larvae belonged to the species *Strongylus vulgaris*, *Strongylus equinus,* and *Triodontophorus* spp.

## 4. Discussion

The ovicidal activity of *P. chlamydosporia* is well known, and its efficacy against the eggs of a large number of nematode species, including *Toxocara canis*, *Ascaris suum*, *Ascaridia galli*, *Trichuris vulpis*, and horse cyathostomins, has been previously reported [20,27,28,29,30]. For this reason, in this study the ovicidal activity of *B. bassiana*, *M. anisopliae,* and *S. brevicaulis* was compared to that of *P. chlamydosporia*. The obtained results confirmed also on donkey cyathostomin eggs the ovicidal activity of *P. chlamydosporia* reported in previous studies. In addition, the reduction of donkey GIS egg hatch caused by *P. chlamydosporia* in this study (62.3%) is similar to that (67–72.8%) previously observed for *P. chlamydosporia* on horse cyathostomin eggs [20]. Moreover, the other three fungal species examined in this study showed inhibiting properties on donkey GIS eggs, comparable to that of *P. chlamydosporia*.

As reported in previous European reports on donkey gastrointestinal nematodes [5,9], also in this study most of donkey GIS infective larvae obtained from fecal cultures were identified as cyathostomins. 

To date, *S. brevicaulis* and, mainly, *B. bassiana* and *M. anisopliae* were known only for their negative effects against arthropods, including many parasites of mammals. Therefore, results from this study are the first report about the inhibiting effects on nematode eggs of these fungal species. The ovicidal effects showed by *B. bassiana*, *M. anisopliae,* and *S. brevicaulis* on donkey GIS eggs could be linked to their ability to degrade chitin.

*B. bassiana* is in fact known as an endophytic, cosmopolitan, and entomopathogenic fungus, and it is used for the biological control of different insects, including *Triatoma sordida*, which is a vector of *Trypanosoma cruzi* [31,32]. *B. bassiana* does not infect humans or other mammals. It is considered a natural insecticide against crop pests [18] and a pathogen for ticks and mites [33]. The dispersion of *B. bassiana* spores is currently studied as a means for the control of *Anopheles* mosquitoes, which are cyclic vectors of *Plasmodium* spp. [34]. When the spores of *B. bassiana* meet the body of susceptible arthropods, they are able to pass through the chitinous exoskeleton and enter the arthropod body, germinate, and develop, using them as a source of nourishment [32]. In this study, *B. bassiana* showed the ability to cause a high reduction of donkey GIS egg hatch (62.6%).

On the other hand, *M. anisopliae* is known as a cosmopolitan fungal species that infects many harmful crop pests, including aphids and beetle larvae [35]. It grows spontaneously in all soils, and it can be a parasite of several arthropods, often leading them to death [32]. Once on the body of susceptible arthropods, the conidia of this fungus germinate, and the emerging hyphae penetrate the chitinous arthropod cuticle. The fungus then develops inside the body of the arthropods, as the chewing louse species *Bovicola bovis* and the mites *Psoroptes ovis* and *Psoroptes cuniculi*, which may die within a few days [36,37]. In this study, this fungus caused about 63% reduction of the hatch of donkey GIS eggs, showing an ovicidal activity comparable to that of *P. chlamydosporia* and *B. bassiana*.

Finally, *S. brevicaulis* is a keratinolytic and chitin-degrading mold widely distributed in the soil, plant materials, skin, and feathers [38]. It is considered a common contaminant species, but it can cause infections in humans, in which it may be responsible for endocarditis [39], onychomycosis [40], and keratitis [41]. *S. brevicaulis* has also entomopathogenic properties [42]. In the present study, *S. brevicaulis* was able to reduce the hatching of donkey GIS eggs by a lower percentage (52%) compared to that of other examined fungi, but this difference was not statistically significant.

## 5. Conclusions

It is already known that the spores of *P. chlamydosporia*, when administered embedded in sodium alginate pellets, may pass through the gastrointestinal tract of the horse without viability alterations, exerting their ovicidal activity on GIS eggs contained in the feces. They are therefore potentially able to considerably reduce the number of infective larvae on pastures [43]. Moreover, considering the ability of *P. chlamydosporia* in altering the eggshell of other horse nematodes, as *Oxyuris equi* and *Parascaris equorum* [43,44], this fungal species is now considered as a potential effective biological control method in horse nematodes. Additionally, this survey encourages further studies aimed to evaluate the potential in vivo use of *P. chlamydosporia* as a biological control method in donkey nematodes.

Moreover, results obtained in this study seem to indicate that mainly *B. bassiana* and *M. anisopliae* may potentially be used in the biological control of donkey nematodes, and further studies aimed to evaluate their inhibiting effects against other equid nematode species are also encouraged. The viability and the lack of pathogenic effects of *B. bassiana* and *M. anisopliae* spores when passing through the equid gastrointestinal tract also need to be evaluated in order to assess their potential applications.
